# Patients’ experiences of managing their rare rheumatic disease

**DOI:** 10.1093/rheumatology/keaf330

**Published:** 2025-06-14

**Authors:** Emma Dures, Celia Almeida, Sadia Janjua, Andrew Hunt, Jen Orme, Peter C Lanyon, Nicola Walsh, Joanna C Robson

**Affiliations:** School of Health and Social Wellbeing, College of Health, Science and Society, University of the West of England—UWE Bristol, Bristol, UK; Rheumatology Department, University Hospitals Bristol and Weston NHS Foundation Trust, Bristol, UK; School of Health and Social Wellbeing, College of Health, Science and Society, University of the West of England—UWE Bristol, Bristol, UK; Rheumatology Department, University Hospitals Bristol and Weston NHS Foundation Trust, Bristol, UK; School of Health and Social Wellbeing, College of Health, Science and Society, University of the West of England—UWE Bristol, Bristol, UK; Rheumatology Department, University Hospitals Bristol and Weston NHS Foundation Trust, Bristol, UK; School of Health and Social Wellbeing, College of Health, Science and Society, University of the West of England—UWE Bristol, Bristol, UK; Rheumatology Department, University Hospitals Bristol and Weston NHS Foundation Trust, Bristol, UK; School of Health and Social Wellbeing, College of Health, Science and Society, University of the West of England—UWE Bristol, Bristol, UK; Rheumatology Department, University Hospitals Bristol and Weston NHS Foundation Trust, Bristol, UK; Nottingham University Hospitals NHS Foundation Trust, Nottingham, UK; School of Health and Social Wellbeing, College of Health, Science and Society, University of the West of England—UWE Bristol, Bristol, UK; School of Health and Social Wellbeing, College of Health, Science and Society, University of the West of England—UWE Bristol, Bristol, UK; Rheumatology Department, University Hospitals Bristol and Weston NHS Foundation Trust, Bristol, UK

**Keywords:** vasculitis, lupus, myositis, systemic sclerosis, Sjögren’s, rare autoimmune rheumatic disease, self-management, views and experiences, qualitative research

## Abstract

**Objectives:**

The rare autoimmune rheumatic diseases (RAIRDs) include SLE (lupus), systemic vasculitis, inflammatory myositis, SSc and sjogren’s disease (SD). The objective of the study is to understand patients’ experiences of living with and managing their RAIRD.

**Methods:**

Participants from the UK with a range of RAIRDs were recruited via social media including patient charity networks. Purposive sampling was used to include a range of participants with different conditions and demographic characteristics. A topic guide was developed with patient partners to guide discussions about health-related quality of life with RAIRDs, including support needs. Focus groups were conducted via online video conferencing, audio-recorded, transcribed, checked and anonymized. Data were analysed thematically by an academic psychologist and rheumatologist.

**Results:**

Twenty-six patients with RAIRDs participated in six focus groups (between three and six people per focus group). The median age was 62 years (range 34–82), 21 (80%) were female and 21 (80%) had a diagnosis longer than the last 2 years. Five themes were identified: managing healthcare systems and health professionals; luck of the draw: variation in access and resources; trustworthy and reliable sources of support; support to live well: core care or an added extra; and dealing with the emotional fallout.

**Conclusion:**

This study found that patients shared experiences regardless of their specific RAIRD, suggesting that a combined intervention could meet their common support needs. Further large-scale work is required, including people who may not usually take part in research, to explore the potential content and structure of such an intervention.

## Graphical abstract

**Figure:**
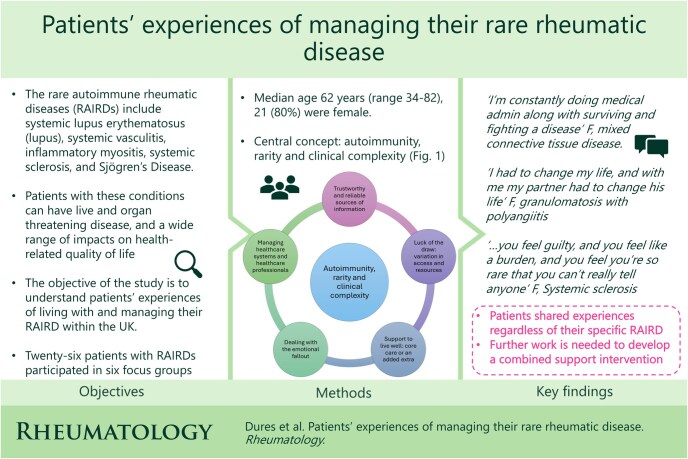

Rheumatology key messages

- Patients from the UK were asked about their experiences of managing autoimmune, rare and clinically complex conditions.
- Shared support needs included dealing with the psychological impact of their rare autoimmune rheumatic diseases and help to negotiate complex healthcare systems.
- Patient themes will inform the development of a larger scale study to develop a combined self-management intervention to improve outcomes and care.

## Introduction

The rare autoimmune rheumatic diseases (RAIRDs) include SLE, systemic vasculitis, inflammatory myositis, SSc and sjogren’s disease (SD) [1]. The RAIRDs are heterogeneous in terms of clinical features ranging from skin rashes, joint pain and mucosal ulceration to major organ involvement, including kidney, lungs and heart failure. The British Society of Rheumatology and the UK Rare Autoimmune Rheumatic Disease Alliance (RAIRDA) patient group propose that these diseases should be considered under a single ‘umbrella’ to increase their profile and improve outcomes [2].

The key challenges facing patients with RAIRDs include managing complex immunosuppressants, chemotherapy and biological medications [3–10]. The RAIRDs can be life and organ threatening: 15–20% of patients with ANCA-associated vasculitis will die within the first year—this is a worse prognosis than breast and prostate cancers [11, 12]. These diseases are chronic and follow a relapsing–remitting pattern that can be difficult to adapt to [2]. There is an impact on social and family life, work, fertility and pregnancy [13–16]. Fatigue is consistently ranked as the most important aspects of health-related quality of life [17]. Having a RAIRD can be very isolating [2, 18, 19] as family, friends and healthcare professionals have often not heard of the individual diseases and psychological support is not routinely provided [20, 21]. Care can be fragmented due to patients being seen by different medical specialties and patients can end up feeling ‘lost in the system’ [2].

A RAIRDA survey of 2300 members highlighted that 61% are struggling to cope with their condition, 45% had reduced or stopped work and 45% felt their health condition had negatively impacted their family [2]. Management guidelines for individual diseases highlight the need for tailored information provision, psychological support, education, support from peers and health professionals, and access to support groups [6–10]. Currently, the support offered to patients with RAIRDs can differ depending on local clinical interest into specific diseases [21].

The National Health Service (NHS) 5-year forward plan highlights self-management as a key for patients with long-term conditions [22]. Self-management aims to empower people with long-term conditions to actively collaborate in their healthcare and management of their condition [23]. A survey of UK health professionals demonstrated that 80% of NHS departments have no self-management programs accessible for patients with RAIRDs [21]. Focus on individual rare diseases may not be feasible as a strategy to support everyone with a RAIRDs across the UK.

The aim of this study was to explore the impact of RAIRDs on patients, including their associated support needs and how to meet them.

## Methods

### Study management

This was a qualitative study with data collected via online focus groups. We selected this design because we were interested in patients’ views and experiences, both shared and divergent. We wanted the flexibility of a semi‐structured format in which participants were asked the same core questions, but we could explore issues in-depth and develop new lines of enquiry based on the group’s responses [24]. The study steering committee including one patient partner oversaw the study and developed a topic guide for the focus groups including four core questions: exploration of the most significant issues that participants were coping with; the resources that were, or would have been, helpful for self-management; the advantages and disadvantages of support aimed at patients with a range of rare rheumatic diseases at the same time; and participants’ views on who should provide support. These questions were asked in the first four focus groups. Data were then analysed and presented back to participants in the remaining focus groups to refine the final themes. We selected focus groups for data collection because they facilitate debate and clarify convergent and divergent views among those taking part [25]. This was important, because we were interested in the extent to which participants with different rare rheumatic diseases perceived similarities and differences in their health-related experiences and associated support needs.

The study was approved by the Faculty Research Ethics Committee in the School of Health and Social Wellbeing at the University of the West of England (UWE) Bristol (REF: HAS.21.11.036).

### Participants

The inclusion criteria were patients living with RAIRDs, aged 18 years or over. Participants were recruited online via social media advertisements including patient charity networks including the Rare Autoimmune Rheumatic Disease Alliance (RAIRDA), Vasculitis UK, Lupus UK, Scleroderma and Raynaud’s UK, Myositis UK and Sjögren’s UK. Potential participants who responded to the adverts were asked their diagnosis and previous treatment to check eligibility. All participants provided written informed consent prior to their focus group.

### Focus groups

The focus groups were conducted via the Zoom platform between May and August 2022 and lasted 60 min. They were facilitated by a non-clinical research associate (C.A.) and a note taker (J.O.), neither of whom had a prior relationship with the participants. At the start, participants were shown three PowerPoint slides of the principal investigator and consultant rheumatologist (J.C.R.), who was not present during the focus groups, giving a pre-recorded 2-min introduction to the study. This was followed by discussions based on the four core questions. The focus groups were audio-recorded, and the audio files transcribed by a university approved service. C.A. checked the transcripts against the original recordings and anonymized them by removing the names of places and people.

### Analysis

The data were analysed manually by a research psychologist (E.D.) who did not know the study participants. E.D. took an inductive thematic approach from an experiential, critical realist perspective, with semantic level coding [26, 27]. The analytical process began with multiple readings of the transcripts to become familiar with the data, then coding segments of the text that related to the research aims. E.D. then grouped together coded segments with a sharing meaning to form subthemes and themes. This was an interpretive and iterative process. J.C.R. reviewed the six transcripts to provide another perspective on the data. Through discussions, E.D. and J.C.R. agreed on a structure and theme labels that reflected their interpretations of patterns in the data.

## Results

A total of 26 participants [21 female (F), 5 male (M)] took part in six focus groups, with between three and six participants in each and a range of RAIRDs (see Table 1 for summary data and Table 2 for individual participant demographics and conditions). The demographic data show the median age was 62 years (range 34–82). Most participants (21/26) identified as White British (English, Welsh, Scottish or Northern Irish) and were educated to at least degree-level (17/26). Participants were located around England and Scotland, with a cluster in the Southwest, where the research team were based. The majority had been diagnosed for over 2 years (21/26) and they reported their disease was active when they took part in the focus group (21/26).

**Table 1.** RAISE study participant characteristics (*N* = 26)

Sex (*n*, %)	
Female	21 (80.76)
Male	5 (19.23)
Age, years	
Median	62
Range	34–82
Ethnicity (*n*, %)	
English/Welsh/Scottish/Northern Irish/British	21 (80.76)
Other White	1 (3.84)
Other Black	1 (3.84)
Caribbean	1 (3.84)
European	1 (3.84)
Mixed White and Asian	1 (3.84)
Education (*n*, %)	
1–4 GCSEs	3 (11.53)
5 GCSEs	3 (11.53)
Bachelors	17 (65.38)
Other	3 (11.53)
Occupation (*n*, %)	
Employed	5 (19.23)
Self-employed	7 (26.92)
Unemployed	2 (7.69)
Disabled	1 (3.84)
Retired	11 (42.30)
Location (*n*, %)	
NW England	1 (3.84)
W Midlands	4 (15.38)
Wales	3 (11.53)
London	3 (11.53)
SW England	10 (38.46)
SE England	3 (11.53)
Scotland	2 (7.69)
Doctor/specialty (patient self-report)	
Rheumatologist	20
GP	7
Respiratory	5
Neurologist	5
Nephrologist	4
ENT	4
Ophthalmology	4
Cardiology	2
Dermatology	2
Colorectal	1
Oral medicine	1
Pain clinic	1
Gynaecology	1
Endocrinology	1
Orthopaedics	1
Gastroenterology	1
Musculoskeletal	1
Haematologist	1
Vasculitis specialist clinic	1
Type of RAIRD	
SLE	4
Primary SD	8
Inflammatory myositis	7
Granulomatosis with polyangiitis	3
Eosinophilic granulomatosis with polyangiitis	1
MCTD	2
Microscopic polyangiitis	1
SSc	1
Undifferentiated vasculitis	2
Secondary SD	8
Diagnosis (*n*, %)	
Within last 2 years	5 (19.23)
Longer than last 2 years	21 (80.76)
Disease activity (*n*, %)	
Active	21 (80.76)
In remission	5 (19.23)

GP: general practitioner; RAIRD: rare autoimmune rheumatic disease; RAISE: Rare Autoimmune SElf-management programme development.

**Table 2.** Individual focus group participant demographics and conditions

ID	Age (years)	Sex	Ethnicity (self-report)	RAIRD
Focus group 1				
PAR 1	60–70	F	English/Welsh/Scottish/Northern Irish/British	Inflammatory myositis, MCTD, SD, lupus, undifferentiated vasculitis
PAR 2	30–40	M	English/Welsh/Scottish/Northern Irish/British	Granulomatosis with polyangiitis
PAR 3	50–60	F	English/Welsh/Scottish/Northern Irish/British	SD, lupus
PAR 4	60–70	F	English/Welsh/Scottish/Northern Irish/British	SD, mixed cryoglobulinemia syndrome
PAR 5	30–40	F	Latina	Lupus
Focus group 2				
PAR 1	50–60	F	European	Granulomatosis with polyangiitis
PAR 2	60–70	F	English/Welsh/Scottish/Northern Irish/British	SD
PAR 3	40–50	M	English/Welsh/Scottish/Northern Irish/British	SD
PAR 4	60–70	M	English/Welsh/Scottish/Northern Irish/British	Inflammatory myositis
Focus group 3				
PAR 1	60–70	F	English/Welsh/Scottish/Northern Irish/British	Granulomatosis with polyangiitis
PAR 2	80–90	F	English/Welsh/Scottish/Northern Irish/British	SD
PAR 3	70–80	F	English/Welsh/Scottish/Northern Irish/British	Inflammatory myositis, SD, Raynaud’s
PAR 4	60–70	F	English/Welsh/Scottish/Northern Irish/British	SD, undifferentiated vasculitis
PAR 5	40–50	F	Any other Black/African/Caribbean background	Inflammatory myositis, SD, lupus, autoimmune progesterone dermatitis
Focus group 4				
PAR 1	50–60	F	English/Welsh/Scottish/Northern Irish/British	SSc, SD
PAR 2	60–70	F	English/Welsh/Scottish/Northern Irish/British	SD
PAR 3	70–80	F	Caribbean	MCTD
PAR 4	60–70	F	English/Welsh/Scottish/Northern Irish/British	SD
Focus group 5				
PAR 1	60–70	F	English/Welsh/Scottish/Northern Irish/British	Eosinophilic granulomatosis with polyangiitis
PAR 2	60–70	M	English/Welsh/Scottish/Northern Irish/British	Inflammatory myositis
PAR 3	60–70	F	English/Welsh/Scottish/Northern Irish/British	SD
PAR 4	30–40	F	English/Welsh/Scottish/Northern Irish/British	Inflammatory myositis, SD
PAR 5	30–40	F	Mixed White and Asian	SD
Focus group 6				
PAR 1	70–80	F	English/Welsh/Scottish/Northern Irish/British	SD
PAR 2	50–60	F	English/Welsh/Scottish/Northern Irish/British	Inflammatory myositis
PAR 3	70–80	M	English/Welsh/Scottish/Northern Irish/British	Microscopic polyangiitis

RAIRD: rare autoimmune rheumatic disease; M: male; F: female; PAR: Participant.

**Figure 1.**
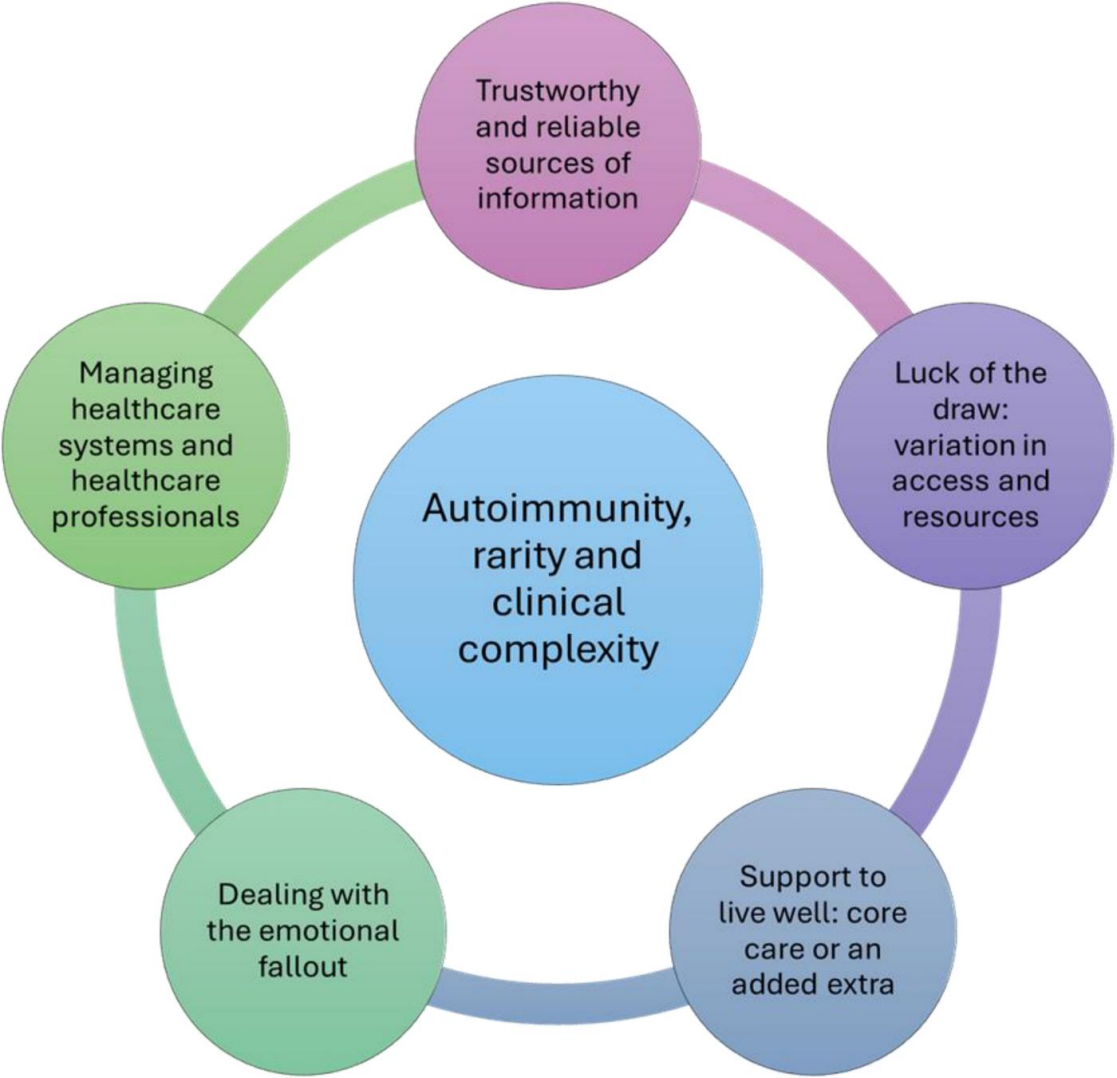
Key themes related to having a rare autoimmune rheumatic disease (RAIRD)

A central organizing concept was identified, linking the entire data set, with five related domains beneath this (see Fig. 1).

### Central organizing concept: autoimmunity, rarity and clinical complexity

Participants discussed how the rarity and clinical complexity of their conditions could make them feel ‘lost and isolated with this illness that keeps getting worse’ (F, SD and undifferentiated vasculitis, age 60–70 years). This sense of being alone was often attributed to family, friends and some health professionals not knowing about RAIRDs. Participants experienced other people’s lack of understanding as disbelief or skepticism about the nature and impact of their health condition, which could exacerbate feelings of vulnerability.

*‘it’s very easy to be made to feel with autoimmune diseases that because your body’s attacking you, that other people are attacking you as well and criticising your choices’* (F, SD, age 50–60 years)

In their experience, their care was more complex than more common rheumatic conditions, such as RA, at every stage, from diagnosis to treatments and long-term management.

*‘…you can go and see a rheumatologist; they’ve never seen a patient like me in their life’* (F, granulomatosis with polyangiitis, age 50–60 years)

This was linked to a sense that less support is offered but also there is less clarity from health professionals about what good and optimal practice comprises.

*‘I’ve had to push so hard for all of it and I think that only happens with very rare conditions because I think the common treatment, for common conditions, there is a very strong and clear pathway’* (F, SD, age 50–60 years)

### Theme 1: managing healthcare systems and health professionals

There was a strong sense of the hard work and ‘admin’ involved in managing their health, often at a time when participants were particularly vulnerable and struggling with the impact of fatigue, described as ‘top of the list of symptoms with us’ (F, inflammatory myositis, SD, age 60–70 years) and ‘just totally relentless’ (F, SSc, age 50–60 years). Although several participants reported high quality and supportive from some individual health professionals, the lack of effective communication between different specialties and healthcare teams could be a source of frustration.

*‘I’ve had very mixed experiences of consultants… but it is the joined-up thing, like my optician referred me originally for eye problems. Got to the consultant and the optician asked the consultant to include me in the letter and he said no, it’s down to the GP, but it was the optician who was the original instigator of this. So, there’s all this lack of joined up…’* (F, inflammatory myositis, age 50–60 years)

This role in managing their healthcare left some participants feeling burdened and unsupported, rather than active collaborators. Participants were often not sure where to go and who to see in the healthcare system and they described being frustrated by having to join up their healthcare because health professionals did not work in a multidisciplinary way.

‘*I’m constantly doing medical admin along with surviving and fighting a disease’* (F, MCTD, inflammatory myositis, lupus, SD, age 60–70 years)

Managing their healthcare took participants’ energy but also relied on their skills and abilities to navigate healthcare systems to get the care that they needed.

*‘…three different hospitals, six different specialists—I’m managing them, and I don’t have time to be a patient’* (F, inflammatory myositis, age 40–50 years)

### Theme 2: luck of the draw: variation in access and resources

This theme drew on personal and contextual factors, including the recognized wider determinants of health, that influence how patients access and engage with care and support.

The sense from the participants was that they were a well-resourced, proactive group who were already part of patient organizations and confident about taking part in research, yet they often struggled to access services and get the support that they required.

*‘…the Rheumatology department there effectively didn’t know what vasculitis was’* (M, granulomatosis with polyangiitis, age 30–40 years)

However, when local provision was unavailable or poor, some of the participants were able to use private healthcare or find alternative healthcare providers in other regions.

*‘I feel really sad that the only way I get a hearing is to pay for it’* (F, lupus, SD, age 50–60 years)

*‘I travel from [X] to [Y] because they seem like they care more than the people I saw in [city]’* (M, granulomatosis with polyangiitis, age 30–40 years)

This led to reflections on those patients living with RAIRDs but with fewer resources, less money and less confidence about how best to manage their health. The groups reflected on issues such as health literacy and the role of sociodemographic factors as well as variation in service provision across the country. Participants were concerned that the time, financial resources and levels of understanding in relation to their disease and its treatment would not be available to everyone with RAIRDs and would exacerbate health inequalities.

*‘We’re all middle class—what about the people who haven’t had the education or help?’* (F, SD, age 60–70 years)

### Theme 3: trustworthy and reliable sources of support

In relation to what is currently available, participants expressed a concern about unreliable, unsafe or misleading information, which was reinforced because the RAIRDs are such complex conditions.

*‘…there might be some misinformation out there as well, which is quite easy to maybe get sucked into’* (F, SD, age 30–40 years)

*‘…people are giving out information which is medical which they shouldn’t be giving’* (F, lupus, age 50–60 years)

Therefore, being seen as trustworthy was an important first step to engaging patients.

*‘…it wasn’t until I discovered Health Unlimited and the Lupus UK forum that I really began to get a grip on what was going on’* (F, lupus, age 50–60 years)

*‘I try not to do a Google search, so you go to sites that you know you can trust. I, I’d always start with the NHS one but then I’d always want to go a bit deeper’* (F, inflammatory myositis, age 70–80 years)

As well as accurate and well-informed support from legitimate sources, patients wanted interactions and materials to be positive and constructive and not to focus too much on difficulties and negative experiences. As such, participants wanted support to have clear aims, good content, a link to theory and a clear set of outcomes.

*‘I don’t like the idea of sitting and just wallowing in the same theme’* (F, MCTD, age 70–80 years)

### Theme 4: support to live well: core care or an added extra?

One of the difficulties participants described in relation to managing their health was that it did not feel legitimate to talk about emotional health during consultations and interactions with health professionals, despite the significant impact of their RAIRD on their lives.

The understanding was that health professionals needed to prioritize the physical health agenda, but that meant participants had few opportunities to talk about their wellbeing or to access mental health and psychological support. In their experience, a holistic or whole person approach was rarely part of their core care.

*‘the last thing they [Drs] want to hear is you saying, I don’t want you to feel my joints, or test my leg strength, I want to talk about the social impact, what this has done to my life, and ways I can, you know, help improve it, they absolutely do not want to talk about that’* (M, inflammatory myositis, age 60–70 years)

These data suggest that there are assumptions, possibly on the part of both patients and health professionals, about which issues it is appropriate to raise. There is potential here for agenda setting and explicit invitations to discuss wellbeing as part of core care.

*‘I get 15 minutes to talk about everything to do with my lung disease and my muscle weakness and my heart condition… so I don’t want to be getting into this stuff about how incredibly fatigued I am, the destruction of my life that this condition has brought about… but then where does it go? I just don’t, there isn’t anywhere to take it’* (F, inflammatory myositis, age 30–40 years)

‘*12½ years of having a long-term condition, not once has any medical professional said to me, how are you really? Are you having difficulty coping mentally?*’ (F, SD, age 60–70 years)

### Theme 5: dealing with the emotional fallout

Participants shared their loss and grief for their previously ‘healthy’ body, including the physical, social and emotional changes they were living with and a changed future that they had not envisaged. It could be overwhelming when participants could not discuss this impact.

*‘…effectively dealing with the grief of losing part of your life or the possibility of part of your life’* (M, granulomatosis with polyangiitis, age 30–40 years)

*‘I terrified myself and not having somebody to go and talk to about it and get it in perspective’* (F, undifferentiated vasculitis, age 60–70 years)

Patients were aware that this emotional fallout was also felt by those around them. This could provoke health-related guilt, a psychological factor that can occur when someone with a long-term condition feels a disconnect between how they are and how they think they should be [28].

*‘I had to change my life, and with me my partner had to change his life’* (F, granulomatosis with polyangiitis, age 50–60 years)

*‘…you feel guilty, and you feel like a burden, and you feel you’re so rare that you can’t really tell anyone’* (F, SSc, age 50–60 years)

## Discussion

This UK qualitative focus group study explores the impact of living with a range of rare rheumatic conditions. Rarity and clinical complexity are highlighted as particular issues shaping patients’ experiences of living with and managing their condition: people can feel isolated and unsupported by health professionals and find it difficult to get reliable information. There are shared needs including dealing with the physical and psychological impact of these long-term and serious diseases as well as navigating complex medication regimens and healthcare systems.

We collected data via focus groups which enabled us to capture collective insights, interactions and reflections in a dynamic and supportive environment [29, 30]. However, there was limited scope to explore individual experiences in great depth compared with one-to-one interviews and this could be a valuable future study [31].

The strength of this study is the range of participants included in terms of condition and geographical location. This was achieved by recruitment via social media, in collaboration with patient charities, who are expert in supporting people with these diseases. The majority of focus group participants had lived with their RAIRD for more than 2 years and were able to draw on their extensive experiences. However, as the participants themselves identified, the sample were generally highly educated, computer literate and active members of patient organizations. We need to explore whether patients with diverse sociodemographic and economic backgrounds have similar experiences and support needs.

Clinical outcomes in the RAIRDs can vary between patients with significant health disparity. For example, in lupus, the young, those of non-European ancestries (Black, East Asian, South Asian and Hispanic) and individuals living in poverty are at risk of worst outcomes [4, 5]. The Office for Health Improvement and Disparities defines health inequality as ‘avoidable differences in health outcomes between groups or populations—such as differences in how long we live, or the age at which we get preventable diseases’ [32]. People living in more deprived areas are at risk of multi-morbidity, with additive mental and physical health problems [33] To overcome these health disparities among disadvantaged populations, their perspectives need to be considered within research. The youngest participant in this focus group study was 34 years old, and over 80% participants were of White British heritage. This is a clear limitation of the study and should be addressed specifically in future research. Potential barriers to inclusive participation in research include proximity to venues for in-person data collection, work and caring responsibilities that limit the hours people are available to take part, or poorer health not permitting individuals to participate, and these should be considered and reflected in the financial and time resources allocated [34]. Lack of funding and organization for translation and interpretation services may act as further barriers, whilst community outreach, relationship building and improved communication may act as enablers to wider participation [34]. We also need to consider that research study processes, such as engaging with Participant Information Sheets and providing written informed consent, might be a barrier to participation for some patients. In addition to further qualitative studies with diverse and inclusive samples, we need to triangulate our findings and examine their generalizability using quantitative methods. A survey design would allow us to make meaningful comparisons in terms of support needs across disease groups, and according to a range of clinical and sociodemographic factors.

This focus group study across the rare rheumatic diseases echoes the findings of the RAIRDA patient questionnaire surveys in terms of highlighting a systemic lack of support for people with RAIRDs and the impact on health-related quality of life [2]. This study also supports the findings of the VOICES (Vasculitis Outcomes In relation to Care ExperienceS) project in vasculitis care, which has identified components including better integration between services, multi-disciplinary team working and specialist nurse support as being protective against poor outcomes including severe infections and hospital admissions [35]. Beyond rheumatology, people with rare diseases in general, defined as a condition which affects <1 in 2000 people [36], are often poorly served across the UK. A 2023 patient survey reported that only 12% had a care coordinator, 32% were seen in a specialist centre and 10% had a care plan—overall only 2% had access to all three key elements [37]. The 2021 UK Rare Disease Framework identified increased awareness of rare diseases by health professionals, better coordination of care and access to timely specialist care as key priorities—all issues raised by the focus group participants in this study [36].

Our 2020 survey of UK health professionals highlighted that 80% of NHS departments had no self-management programmes accessible for patients with RAIRDs [22]. Patient self-management programmes in RA improve wellbeing and reduce healthcare utilization [38–41]. Self-management programs for people with RA usually exclude patients with RAIRDs; possibly due to the perception that these diseases may be too complex or different to manage in that setting [21], yet their needs are as great [3]. A systematic review of six randomized controlled trials of self-management programmes in SLE demonstrated improvements in anxiety, depression, stress and disease activity [42]. There are no randomized controlled trials of self-management or educational programmes in vasculitis, myositis or SSc. In SSc, pilot studies of self-management programs appear to improve self-efficacy including in pain management [43, 44].

There is a need for dedicated psychological support and self-management programmes for people with RAIRDs, a whole-team approach (specialist teams empowering people to manage their own care), staff training (e.g. brief psychological interventions) and signposting to resources, including patient charities [21]. It is, however, important that patients feel informed and confident and that the healthcare system is there to support them in this process, so they know that they can access the right care at the right time as needed. More than 80% of rheumatology health professionals reported they would welcome additional training in self-management and psychological support for people with RAIRDs [21].

Owing to the rarity of these RAIRDs and current lack of formal psychological support, there is an opportunity to develop a combined cross-condition self-management intervention to address the common impacts of these conditions, reduce isolation, improve health outcomes and be more economically viable for the NHS than disease-specific provision. This could be designed to be feasible and acceptable for different types of rheumatology unit in the UK to deliver as part of routine care. If co-designed with patients and health-professionals, such an intervention could potentially increase health professional engagement and confidence in support for patients with RAIRDs across all types of specialist and district general hospital settings. To explore this initiative further, more work to engage with people who do not usually take part in research and a wider and more in-depth exploration of the key components and delivery methods is required.

## Data Availability

The data underlying this article are available in the article.
